# Expert Searching in the Google Age

**DOI:** 10.5195/jmla.2018.347

**Published:** 2018-01-02

**Authors:** Carolyn G. Biglow

This book is an excellent resource for health sciences librarians, whether they are experienced searchers or new librarians. The experienced librarian will be able to use Jankowski’s tips as a refresher course, and the new librarian can use the book as a brief introduction to searching. In either case, librarians will appreciate the opportunities to practice and improve their search skills by using the examples and exercises that the author supplies.

Starting the book with a sample search request provides a very useful way of introducing the subject of database searching. Jankowski also ends many of the eight chapters with a section called “Apply the Learning.” These sections contain lists of tasks that illustrate the subject covered in each chapter. Opportunities to create a population, intervention, comparison, outcome (PICO) question or to find other databases to search are very helpful because they allow practicing and understanding concepts that are often not offered in library school.

Learning to do a reference interview is covered in the second chapter of the book. This chapter is a valuable resource for any librarian who is new to the back-and-forth that is required when trying to form a search strategy with a requestor. A section on “Questions to Ask” includes examples of how the searcher can zero in on the specifics of the search strategy that is being formed. This chapter also discusses the good and bad aspects of PICO questions and how to determine the level of collaboration that is needed with the search requestor.

Some readers might expect a longer book, considering the subject matter that is covered. There are some subject areas that could use more coverage (perhaps in a future edition), but Jankowski’s examples and activities provide a primer for any novice or expert searcher to improve and refresh their searching and teaching skills. Most importantly, Jankowski makes a very good case for supporting the value that librarians have as expert searchers, whether utilizing medical databases or Google.

